# Differential metabolomics networks analysis of menopausal status

**DOI:** 10.1371/journal.pone.0222353

**Published:** 2019-09-18

**Authors:** Xiujuan Cui, Xiaoyan Yu, Guang Sun, Ting Hu, Sergei Likhodii, Jingmin Zhang, Edward Randell, Xiang Gao, Zhaozhi Fan, Weidong Zhang

**Affiliations:** 1 School of Pharmaceutical Sciences, Jilin University, Changchun, P.R. China; 2 Department of Pharmacy, Daqing Oil-Field General Hospital, Daqing, China; 3 Discipline of Medicine, Faculty of Medicine, Memorial University, St. John’s, NL, Canada; 4 Department of Computer Science, Memorial University, St John’s, NL, Canada; 5 BC Provincial Toxicology Centre, Provincial Health Services Authority, Vancouver, British Columbia, Canada; 6 Department of Laboratory Medicine, Faculty of Medicine, Memorial University, St. John’s, NL, Canada; 7 College of Life Sciences, Qingdao University, Qingdao, China; 8 Department of Mathematics and Statistics, Memorial University, St. John’s, NL, Canada; 9 Discipline of Genetics, Faculty of Medicine, Memorial University, St. John’s, NL, Canada; Universitair Medisch Centrum Utrecht, NETHERLANDS

## Abstract

Menopause is an endocrine-related transition that induces a number of physiological and potentially pathological changes in middle-aged and elderly women. The intention of this research was to investigate the influence of menopause on the intricate relationships between major biochemical metabolites. The study involved metabolic profiling of 186 metabolic markers measured in blood plasma collected from 120 healthy female participants. We developed a method of network analysis using differential correlation that enabled us to detect and characterize differences in metabolites and changes in inter-relationships in pre- and post-menopausal women. A topological analysis was performed on the differential network that uncovered metabolite differences in pre-and post-menopausal women. In this analysis, our method identified two key metabolites, sphingomyelins and phosphatidylcholines, which may be useful in directing further studies into menopause-specific differences in the metabolome, and how these differences may underlie the body's response to stress and disease following the transition from pre- to post-menopausal status for women.

## Introduction

The transition into menopause induces significant developments in a number of organ systems in the body, as well as the skeletal system. These developments could lead to pathological and physiological changes [1,2]. For example, the prevalence of osteoarthritis (OA) is higher among women than among men, and the difference in prevalence further increases after menopause. This could indicate the significant role played by female sex hormones in the etiology of musculoskeletal degenerative diseases [3,4]. During our investigations, many of the metabolites examined in study participants affected by OA were found to show associations related to both age and gender [5,6]. Considering that changes in organ biochemistry accompany the development of conditions such as heart disease, premature mortality, and OA, disturbances in metabolism are either a simple consequence of pathological changes or factors that contribute to the development of disease, or a combination of the two. Considering its effects on biochemical metabolism, menopause, combined with numerous other influences such as genetics and the environment, may play a role in the development and outcome of a disease. Therefore, establishing metabolic biomarkers related to menopause will enable us to understand these influences and their biological implications.

Metabolomics, as a method of analysis, allows for the investigation of underlying mechanisms that control biological functions and the expression of various phenotypes through the involvement of studies that investigate the various states and conditions of large groups of metabolites [6,7]. The analysis of data from these studies, using techniques that allow binary class discriminations such as partial least square discriminant analysis (PLS) and principal component analysis (PCA), often reveal complex relationships between metabolites and phenotypes [8]. When studying the effects of these metabolites on intricate physiological states, however, the regulatory systems in which they function need to be taken into consideration [9]. These regulatory systems can provide the cellular context of all metabolites of interest, as well as a means of identifying dysfunctional subnetworks in each disease or physiological state. However, due to the limited availability of accepted methodologies, those types of analyses are not frequently used [10]. The lack of established procedures for the analysis of metabolite correlations, for example, has caused it to see only limited adoption. If such correlations were to be investigated, however, the results would be significantly interesting and could reveal information about complex biochemical systems and their connections.

Our primary goal in this work was to advance a method for determining how pairs of metabolites that exhibit significant differential correlations during pre- and post-menopause are interconnected, with the ultimate objective being the investigation of the effects of menopause on the components of metabolic makeup and the complex relationships between those components. This specific method is referred to as the differential correlation network approach, and the methodology used is markedly distinctive from current methods of analysis. By applying the method of topological analysis of differential associations, we can identify the metabolites that have significant influence in controlling information flow and network functional connectivity.

## Patients and methods

### Patients

The study participants were adult female volunteers recruited from the Newfoundland population. The present study was part of ongoing CODING (Complex Diseases in the Newfoundland Population: Environment and Genetics) study that was initiated in 2003 [11]. The inclusion criteria for the cohort were as follows: a) adults between the ages of 21and 76 years old; b) 3-rd or higher generation Newfoundlander; and c) not pregnant at the time of study. A total of 120 healthy women were randomly selected from the overall sample. The mean age of subjects was 50±12.8 years. The mean BMI was 29.2±5.6 kg/m^2^. Only participants without liver, renal, metabolic or inflammatory diseases were recruited. The general characteristics of the subjects were shown in **Table 1**. Information concerning menopausal status was obtained through a questionnaire on menstrual history. In total, 55 women aged 21 to 54 years were deemed as pre-menopausal after reporting regular menstruation, while the remaining 64 women aged 40 to 76 years were designated as post-menopausal after reporting periods of amenorrhea longer than 12 months. Medical information was gathered from the participants using a self-administered questionnaire. All methods were performed in accordance with the relevant guidelines and regulations, and the study protocol received approval from the Health Research Ethics Authority of Newfoundland and Labrador. Written informed consent was obtained from all of the volunteers.

**Table 1:** Characteristics of the study participants in the pre- and post-menopause.

Variables	Pre-menopause	Menopause	*P*-value
**Age (years)**	39.4 ± 9.1	57.2 ± 8.5	1.76×10^−19^
**BMI (kg/m**^**2**^**)**	29.4 ± 7.2	28.3 ± 4.8	0.351
**Physical activity level**	8.2 ± 1.5	7.5 ± 1.6	0.020
**Age of menarche**	12.4 ± 1.4	13.2 ± 2.1	0.027

### Demographics and anthropometrics

Demographic information was extracted from the self-administered questionnaire. All anthropometric measurements were taken in the morning hours after a 12-hour period of fasting. Participants were weighed to the nearest 0.1kg using Health O Meter scale (Bridgeview, IL). A fixed stadiometer was used to measure participants' height to the nearest 0.1 cm. Weight and height measurements were used to calculate BMI, which is expressed in kilograms per square meter. Age was determined at the time of blood collection.

### Plasma sample preparation

Whole blood samples were collected after at least 8 hours of fasting using commercial EDTA tubes (lavender tops). Plasma was separated within 15 min of collection using the standard protocol of centrifuging at 2,000 ×g for 10 mins. The separated plasma was then immediately transferred into a polypropylene storage container and stored at -80°C until analysis. The specimen storage time was less than two years for all samples. Sample preparation was according to the laboratory workflow (**S1 Fig**).

### Metabolomics data collection

We performed metabolic profiling in plasma samples using the Waters XEVO TQ mass spectrometry system (Waters Limited, Mississauga, Ontario, Canada), combined with the Biocrates Absolute IDQ p180 kit. This enabled the measurement of 186 metabolites including 90 glycerophospholipids, 40 acylcarnitines (including free L-carnitine), 21 amino acids, 19 biogenic amines, 15 sphingolipids, and 1 hexose (> 90% is glucose). The complete list of 186 metabolites is provided in the **S1 Table**. The metabolic profiling method used in this study was previously described [12]. Briefly, acylcarnitines, glycerophospholipids, and sphingolipids were analyzed on the system by flow injection analysis (FIA) and using positive ion electrospray ionization. Hexose was analyzed on a subsequent FIA analysis and using negative ion electrospray ionization. Amino acids and biogenic amines were analyzed using an ACQUITY UPLC System connected to the Waters XEVO TQ mass spectrometry system and using positive ion electrospray ionization **(S2 Table)**. Identification and quantification of metabolites was achieved using internal standards and by multiple reaction monitoring (MRM) methodology. Data analysis and calculation of the metabolite concentrations, analyzed by FIA (acylcarnitines, glycerophospholipids, sphingolipids, and hexoses), was automated using the MetIDQ software (BIOCRATES Life Sciences AG). Analysis of peaks obtained by UPLC (amino acids and biogenic amines) was performed using the TargetLynx Application Manager software, and the results were then imported into the MetIDQ software for further processing and statistical analysis.

### Statistical methods

#### Measurement of metabolic profiling

Metabolite concentrations in plasma samples were measured using mass spectrometry as described above. Metabolites that were present at measurable levels in at least 80% of samples were selected for analysis. In samples where the levels of metabolites were below detection limits a concentration equivalent to half of the minimum quantified level was assigned so that further analysis, requiring quantitative data, could occur. Overall, in all plasma samples comprising the dataset, 168 of the 186 metabolite panel were successfully quantified.

Prior to any analysis, we performed covariant adjustment based on participant age, BMI, physical activity, and age of menarche, in order to remove any confounding associations. The covariate adjustment was done through regressing the levels of metabolites on the potential confounding factors. The residuals of the regression were used in further analysis. Imputation was performed to fill missing data entries (missing rate less than 5% per metabolite) with the population average. Data were further normalized to a mean of 0 and unit standard deviation.

#### Differential correlation network analysis of key menopause-associated metabolites

Metabolite concentrations may correlate due to the complex cascading biochemical reactions in metabolism. The correlation may or may not associate with phenotype. Therefore, differential correlation analysis allows computing the change of the correlations of metabolite pairs in different phenotypic groups [13,14]. Moreover, networks provide a global overview and analytical tool to investigate the relationships between a large number of different entities, and can be used to characterize the differential correlations between multiple metabolites [13].

As described in our previous study [14], Pearson’s correlation coefficient r was used to evaluate the correlation between a pair of metabolites in subjects who were pre-menopausal and post-menopausal. The correlation coefficients *r*_pre_ and *r*_post_ were used to evaluate the change in correlation between two metabolites in each of the two physiological categories defined as the pre-menopausal and post-menopausal. Namely, for metabolites *i* and *j*, the differential correlation *r*_diff_ (*i*, *j*) was determined as the normalized difference of Fisher’s z-transformations of *r*_pre_(*i*, *j*) and r_post_ (*i*, *j*),

$$r_{diff}(i,j)=\sqrt{\frac{n_{pre}-3}{2}}\times z_{pre}(i,j)-\sqrt{\frac{n_{post}-3}{2}}\times z_{post}(i,j)$$ (1)

In which z is the Fisher’s z-transformation of the correlation coefficient r,

$$z_{pre}(i,j)=\frac{1}{2}ln[\frac{1+r_{pre}(i,j)}{1-r_{pre}(i,j)}],z_{post}(i,j)=\frac{1}{2}ln[\frac{1+r_{post}(i,j)}{1-r_{post}(i,j)}]$$ (2)

We used *n*_pre_ and *n*_post_ to denote the total amount of samples in the pre-menopause and post-menopause categories. The differential correlation figure indicates a change in the normalized correlation between the two distinct categories. By applying this approach, we can determine whether any two corresponding metabolites are differentially correlated in the pre-menopausal and the post-menopausal groups of subjects. We used a 1×10^3^ fold permutation test to assess the levels of significance of the differential correlations.

## Results

### Metabolite correlations in pre-menopause and post-menopause

Overall, we calculated the pairwise Pearson's correlations of 168 metabolites in samples from pre- and post-menopausal women. The majority of 13,861 pairs of metabolites were positively correlated as observed in both pre- and post-menopause cases and controls. In order to determine the significance of pairwise correlations, we employed Bonferroni multiple-testing correction and used a p-value threshold of 0.05. About 80% of all correlated pairs in pre-menopausal women were also determined to be correlated in post-menopausal women. Considering that there was a significant overlap in metabolite correlations between the two phenotypic conditions, it can be inferred that the majority of correlations were caused by "housekeeping" biological reactions and that they were unrelated to menopausal status.

### Differentially correlated metabolites between pre-menopause and post-menopause

**Fig 1:**
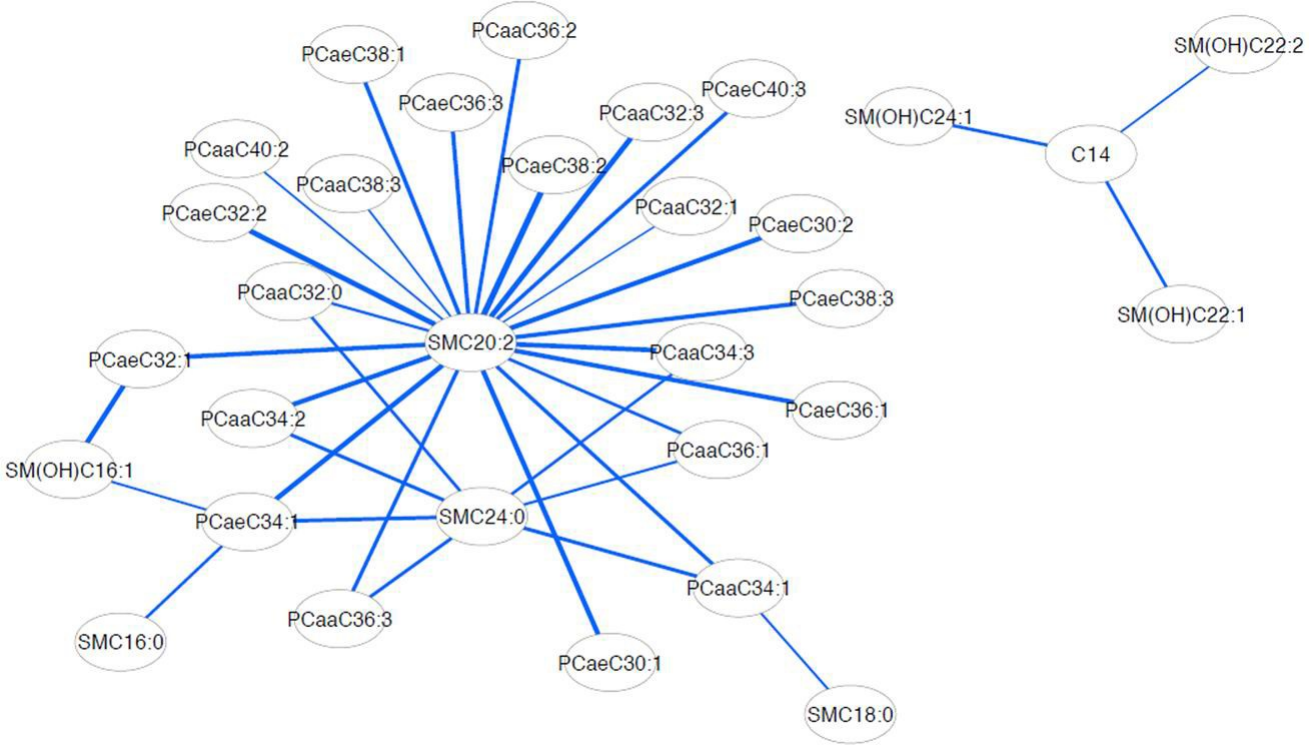
The differential correlation network showing linkages between components of the metabolite dataset. (Only pairs that have significant differential correlations are shown. The network is visualized using the force-directed layout presentation with a closer node layout distance representing a stronger pairwise correlation. Edge width is proportional to differential correlation strength and edge color (blue) shows that all the differential correlations are negative).

On comparison between pre- and post-menopausal women, 829 metabolite pairs showed differential correlations with a significance level of permutation testing p ≤ 0.05, and 155 metabolite pairs with a level of p ≤ 0.01 **(S3 Table**). We used these 155 pairs of metabolites to build the differential correlation network for pre- and post-menopausal subjects. All of the metabolite pairs had negative differential correlations, denoted by the blue edges in the graph as shown in (**Fig 1**). The node degree of the sample network had a mean of 4.05, in which SM (OH) C14:1 had a core status with a degree of 19, showing how robust the information flow and connectivity were in the network (**S4 Table**).

### Differential metabolites between pre-menopause and post-menopause

**Fig 2:**
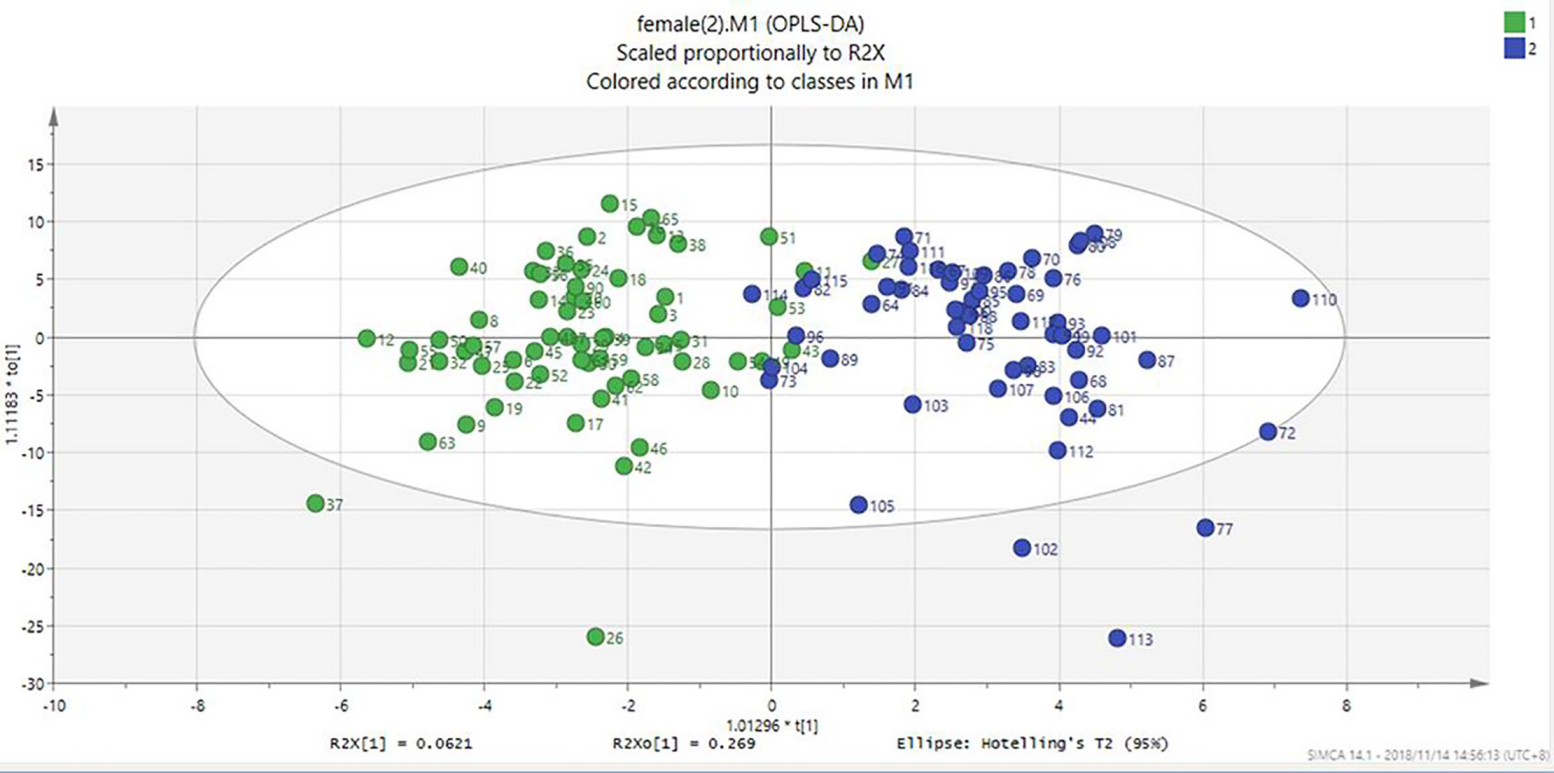
Scores plots of the OPLS-DA analysis of the metabolic profiles in plasma of women before- and post-monopause.

**Fig 3:**
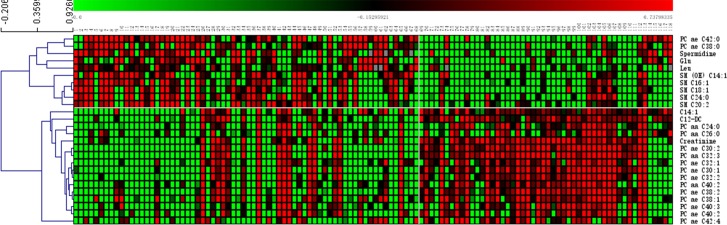
Heatmaps of significant metabolites for the separation of the pre-menopausal and menopausal groups.

We also analyzed the results of the plasma metabolite profiles of subjects from the pre- and post-menopausal groups using the OPLS-DA method. The two groups separated unambiguously with the Q2 = 0.54 (**Fig 2**) using VIP>1 and p value <2.9×10^−4^ (0.05/168) as criteria, 26 metabolites, including 15 glycerophospholipids, 5 sphingolipids, 2 amino acids, 2 biogenic amines and 2 acylcarnitines were identified as key metabolites for the separation of the pre-menopausal and menopausal groups (**Fig 3**). Of these, most were glycerophospholipids (13/15), the 2 acylcarnitines were at higher levels in the pre-menopausal group, and all of the sphingolipids and 2 amino acids were at higher levels in the post-menopausal group. Additionally, S-plot analysis was used to test the identified metabolites. The S-plot model indicated that the values of metabolites were between 0.17 and -0.18. These values were mainly distributed at both ends in the S-plot model loadings, and were consistent with the VIP generated by the OPLS-DA.

Linear regression analysis was used to examine the association between identified metabolite (26 metabolites) concentrations and menopause using covariant-adjusted metabolites. Finally, 3 metabolites were identified as associated with menopause. These metabolites were leucine (p = 0.0016), PC ae C42:0 (p = 0.005) and PC ae C38:0 (p = 0.024).

## Discussion

Existing metabolomic studies in menopause have revealed relationships with certain metabolic changes [15,16]. Using an NMR-based platform, Auro *et al*. found that menopause was associated with changes in the levels of several amino acids, total fatty acid levels and monounsaturated, omega-7 and -9 fatty acids [15]. Similarly, Yamatani *et al*. reported that significantly higher levels of fatty acid metabolites were found in the visceral fat in post-menopausal women compared with pre-menopausal women [16]. In addition to analyses using absolute concentrations of metabolites, changes in the relationships between menopause-associated metabolite pairs were analyzed for the first time in this study. By employing the creation and analysis of the differential correlation network, the network’s core metabolites could be identified. The metabolites identified in this way were closely related to the correlational changes associated with menopause. As described in the Methods section, the differential correlations of the metabolite pairs were calculated by comparing their correlations in pre-menopausal and post-menopausal groups. Subtracting correlations found in pre-menopausal women from those who were post-menopausal enabled us to magnify differentially correlated pairs of metabolites, while negating the correlations that were present in both categories. As can be seen in **Fig 1**, all of the differential correlations had negative signs, indicating that the correlations between the menopause-associated pairwise metabolites were decreasing. We were able to put emphasis on the correlations that were associated specifically with changes in the physiological state by using the differential correlation method. Our investigation yet again demonstrated how powerful network analysis can be for characterizing the complex relationships between entities, in this case increasing the extent to which we understand metabolic changes induced by menopause. A number of metabolites that play important roles in modulating connectivity through the network, as well as network information flow were revealed through topological analysis on node importance using centrality measures. In the core of the network, the hub-and-bottleneck metabolites were sphingomyelins, specifically SM (OH) C14:1, while the metabolites present on the peripheral are mostly phosphatidylcholines and acylcarnitines. Furthermore, plasma metabolite profiles of subjects from the pre- and post-menopausal groups were also analyzed using the OPLS-DA method. The two groups separated unambiguously with the Q2 = 0.54 (**Fig 2**). Using the criteria of VIP>1 and p value <2.9×10^−4^(0.05/168), 26 metabolites, including 15 of 26 glycerophospholipids, 5 of 26 sphingolipids were identified as the key metabolites for the separation of pre-menopausal and post-menopausal groups. After adjustment for age, BMI, physical activity and age of menarche, 3 of 26 metabolites (leucine, PC ae C42:0 and PC ae C38:0) are still significantly associated with menopause.

Sphingomyelins control membrane fluidity and promote signal transduction, making them important components of cell membranes, particularly in neuronal cells. The plots were drawn smoother to further define age-related changes in metabolite concentrations in women and men. We found that the concentrations of SM C20:2 and SM C24:0 increased sharply after 40 years of age. Interestingly, in our separate study in the males (unpublished observation), sphingomyelins were not significantly associated with age, which showed that the changes in sphingomyelins were female specific and possibly menopause-dependent. The higher levels of sphingomyelins in older women were consistent with Yu *et al*’s research [17]. In a more recent study, global lipid profiles were compared with associated mRNA levels of the proliferating and replicative senescent BJ fibroblasts. The changes in lipid composition of cells that were most significant during senescence were identified in Sphingolipids [18]. Phosphatidylcholines (PCs) are the most abundant class of phospholipids. It incorporates choline as a head group and mainly resides in the outer layer of the cellular membrane. Nearly 80% of men and post-menopausal women developed liver or muscle damage when deprived of PCs, whereas only 43% of pre-menopausal women developed similar organ damage [19–21]. Sphingomyelins and phosphatidylcholines are both components of plasma lipoproteins and are involved in lipoprotein assembly, and show association with menopausal status. During menopause, the heightened number of lipoproteins likely reflects how changes in hormone levels influence liver enzymes [22,23], but these changes might also relate to weight gain and insulin resistance [24,25].

In addition, our study also demonstrated that menopause contributes to the metabolic composition of body fluids and could act as a confounder in other metabolomic investigations. In future studies, changes in metabolite levels and relationships occurring as a result of menopausal status should be adjusted in metabolomics research to avoid any false discoveries.

## Conclusion

This study investigated menopause from the metabolic perspective. To the best of our knowledge, this was the first study that used the differential correlation metabolomics approach to classify participants with pre- and post-menopause state. The metabolic profiling reflects directly what is happening in pre-and post menopause and yields the most accurate and real-time metabolic profile that is relevant to menopause. We studied differential correlation of pairwise metabolites in women pre-menopause and post-menopause, and identified a set of metabolites that were significantly associated with menopause. On progression to menopause, women experience unique changes in the metabolism of SMs, PCs, acylcarnitines and amino acids that are significantly different from pre-menopausal women. The findings of this study furthers our understanding of metabolomic changes induced by menopause. These findings will be of value to future studies investigating the effects of menopause on health and towards diminishing the adverse metabolic effects during post-menopausal life.

There are some limitations. First, we did not have detailed dietary and drug used information on the study participants, which might have had an influence on metabolite concentrations. Second, our sample size was modest and a follow-up study with a large sample size is required to verify these findings. Third, we used a targeted metabolomics approach, thus, we might have missed important menopause-associated metabolites which we were unable to measure.

## Supporting information

S1 Fig Overview of laboratory workflow. (DOCX)

S1 Table List of metabolite concentrations determined using the Biocrates 19 AbsoluteIDQ kit. (DOCX)

S2 Table The gradient, collision energy and mode of LC-MS analysis. (DOCX)

S3 Table Signifificant pairs of metabolites showed the strong negative differential correlation. (XLSX)

S4 Table Metabolites as hubs (high degree) and bottlenecks (high betweenness or closeness) in the network. (DOCX)
