# Supplementary figures and images for: Differential metabolomics networks analysis of menopausal status
Source: PLoS One. 2019 Sep 18;14(9):e0222353. doi: 10.1371/journal.pone.0222353 (PMC6750885; doi:10.1371/journal.pone.0222353)

**Supplementary Figure 1** overview of laboratory workflow


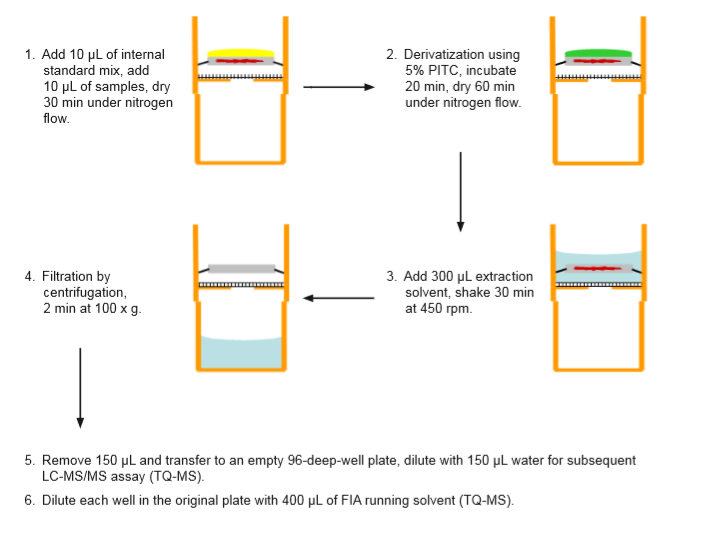

Supplement: S1 Fig — (DOCX) [file pone.0222353.s001.docx]

**Supplementary Table 2.** The gradient, collision energy and mode of LC-MS analysis


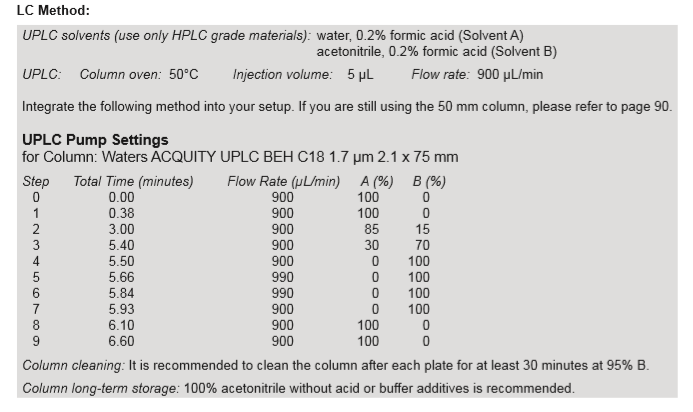


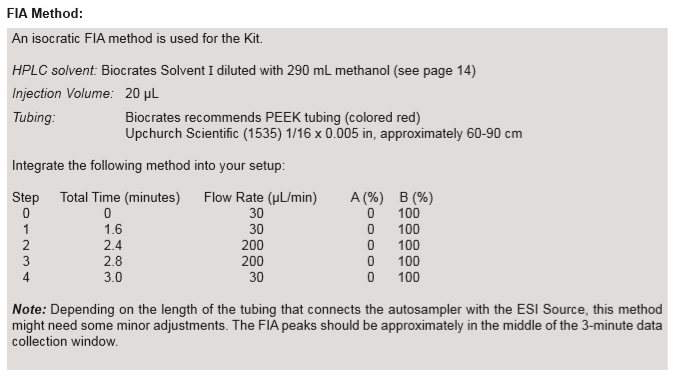


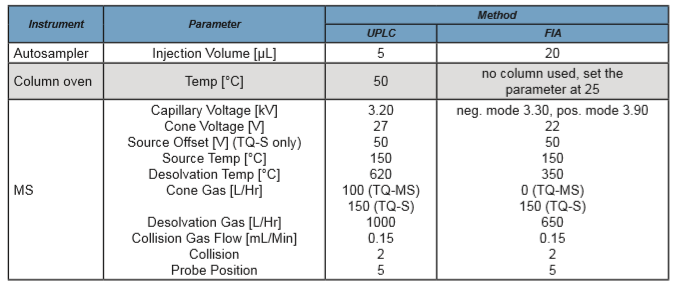

Supplement: S2 Table — (DOCX) [file pone.0222353.s003.docx]
