# Supplementary material for: Differential metabolomics networks analysis of menopausal status
Source: PLoS One. 2019 Sep 18;14(9):e0222353. doi: 10.1371/journal.pone.0222353 (PMC6750885; doi:10.1371/journal.pone.0222353)
Supplement: S4 Table — (DOCX) [file pone.0222353.s005.docx]

Supplementary Table 4

Metabolites as hubs (high degree) and bottlenecks (high betweenness or closeness) in the network.

| Name | BetweennessCentrality | ClosenessCentrality | Degree |
| --- | --- | --- | --- |
| SM (OH) C14:1 | 0.263184 | 0.414201 | 19 |
| PC ae C38:1 | 0.121742 | 0.378378 | 14 |
| PC aa C32:1 | 0.341366 | 0.424242 | 13 |
| PC ae C30:1 | 0.075615 | 0.374332 | 12 |
| PC ae C38:2 | 0.094084 | 0.380435 | 12 |
| SM C24:0 | 0.078668 | 0.366492 | 12 |
| lysoPC a C14:0 | 0.22539 | 0.334928 | 11 |
| SM C16:1 | 0.036695 | 0.362694 | 11 |
| SM (OH) C16:1 | 0.031394 | 0.360825 | 11 |
| PC aa C34:1 | 0.06244 | 0.336538 | 11 |
| SM C18:1 | 0.026494 | 0.358974 | 10 |
| SM C16:0 | 0.026494 | 0.358974 | 10 |
| SM (OH) C22:2 | 0.028605 | 0.351759 | 8 |
| PC ae C30:2 | 0.007042 | 0.321101 | 8 |
| C14 | 0.118261 | 0.304348 | 7 |
| SM (OH) C22:1 | 0.24568 | 0.382514 | 7 |
| PC aa C32:0 | 0.005016 | 0.318182 | 7 |
| PC aa C32:3 | 0.024694 | 0.318182 | 7 |
| PC ae C32:1 | 0.003102 | 0.3125 | 6 |
| PC ae C32:2 | 0.003057 | 0.309735 | 6 |
| SM (OH) C24:1 | 0.055366 | 0.339806 | 6 |
| SM C18:0 | 0.0103 | 0.330189 | 6 |
| SM C20:2 | 0.057929 | 0.262172 | 5 |
| PC aa C36:1 | 0.023565 | 0.315315 | 5 |
| PC ae C36:1 | 0.008373 | 0.338164 | 4 |
| lysoPC a C28:0 | 0.137888 | 0.258303 | 4 |
| PC ae C34:1 | 0.005847 | 0.334928 | 4 |
| Spermidine | 0.084472 | 0.209581 | 4 |
| PC aa C38:4 | 0.101863 | 0.318182 | 3 |
| PC ae C36:2 | 0.056431 | 0.290456 | 3 |
| lysoPC a C20:3 | 0.056729 | 0.24911 | 3 |
| PC aa C38:5 | 0.156522 | 0.324074 | 3 |
| PC aa C34:2 | 0.011283 | 0.309735 | 3 |
| C16:2 | 0.005631 | 0.286885 | 2 |
| PC aa C42:5 | 6.14E-04 | 0.301724 | 2 |
| C7-DC | 1 | 1 | 2 |
| PC aa C36:6 | 0.028571 | 0.209581 | 2 |
| PC ae C40:3 | 0.028571 | 0.227273 | 2 |
| PC ae C38:4 | 1.95E-04 | 0.288066 | 2 |
| Cit | 0.001636 | 0.29661 | 2 |
| PC aa C28:1 | 2.09E-04 | 0.254545 | 2 |
| PC aa C40:6 | 0.076736 | 0.338164 | 2 |
| PC ae C40:4 | 1.95E-04 | 0.288066 | 2 |
| PC aa C30:0 | 0 | 0.269231 | 1 |
| C14:1 | 0 | 0.173697 | 1 |
| PC aa C40:2 | 0 | 0.254545 | 1 |
| PC ae C38:3 | 0 | 0.275591 | 1 |
| PC aa C34:4 | 0 | 0.666667 | 1 |
| PC ae C38:0 | 0 | 0.2 | 1 |
| lysoPC a C28:1 | 0 | 0.242215 | 1 |
| C16-OH | 0 | 0.234114 | 1 |
| C18 | 0 | 0.234114 | 1 |
| PC aa C38:6 | 0 | 0.294118 | 1 |
| C0 | 0 | 0.251799 | 1 |
| C6:1 | 0 | 0.251799 | 1 |
| PC aa C42:4 | 0 | 0.173697 | 1 |
| PC ae C34:0 | 0 | 0.185676 | 1 |
| C12 | 0 | 0.173697 | 1 |
| C12-DC | 0 | 0.173697 | 1 |
| C16:1 | 0 | 0.234114 | 1 |
| C5:1 | 0 | 0.251799 | 1 |
| C5-OH (C3-DC-M) | 0 | 0.251799 | 1 |
| C9 | 0 | 0.251799 | 1 |
| PC aa C40:5 | 0 | 0.2 | 1 |
| Trp | 0 | 0.294118 | 1 |
| Arg | 0 | 1 | 1 |
| lysoPC a C18:2 | 0 | 1 | 1 |
| C4:1 | 0 | 0.251799 | 1 |
| C5:1-DC | 0 | 0.251799 | 1 |
| PC ae C36:0 | 0 | 0.294118 | 1 |
| PC ae C40:6 | 0 | 0.294118 | 1 |
| Ala | 0 | 0.666667 | 1 |
| C10:2 | 0 | 0.251799 | 1 |
| C14:2 | 0 | 0.234114 | 1 |
| PC aa C40:4 | 0 | 0.205882 | 1 |
| PC aa C34:3 | 0 | 0.294118 | 1 |
